# Unraveling the Controversy: The Causal Link Between Osteoarthritis and Alzheimer's Disease

**DOI:** 10.1002/brb3.70455

**Published:** 2025-03-23

**Authors:** Jingkai Di, Yujia Xi, Yaru Liu, Likun Qi, Tingting Chen, Shuai Chen, Chuan Xiang

**Affiliations:** ^1^ Department of Orthopedics Second Hospital of Shanxi Medical University Taiyuan China; ^2^ Shanxi Medical University Taiyuan China; ^3^ The Second Hospital of Shanxi Medical University Taiyuan China; ^4^ The Third Hospital of Shanxi Medical University Taiyuan China; ^5^ The Fifth Hospital of Shanxi Medical University Taiyuan China

**Keywords:** Alzheimer's disease, causal relationship, Mendelian randomization, osteoarthritis

## Abstract

**Objectives:**

Research on osteoarthritis (OA) and Alzheimer's disease (AD) is currently highly controversial, and the upstream and downstream relationships between them remain unclear. This study aimed to assess the association between OA and AD using Mendelian randomization (MR).

**Method:**

Summary data from genome‐wide association studies (GWAS) were obtained for OA and AD. Single nucleotide polymorphisms (SNPs) were selected as instrumental variables (IVs), and significant (*p* < 5.0 × 10^−8^) and independent (*r* ^2^ < 0.001) SNPs were extracted for two‐sample MR analyses. Inverse variance weighting (IVW) was used to assess these causal relationships, and meta‐analysis was used to combine MR results from multiple IVWs. Confounders were assessed by multivariate Mendelian randomization (MVMR). Results were reported as odds ratios (OR). Heterogeneity was then tested using Cochran's *Q* test, multiplicity was tested using the MR‐Egger intercept and MR‐PRESSO, and sensitivity analyses were performed using the leave‐one‐out sensitivity test.

**Results:**

The MR results showed a positive causal effect of AD and OA (IVW OR = 19.89, 95% CI = 2.90–136.57, *p* = 0.002; OR = 1.28, 95% CI = 1.11–1.47, *p* = 0.017; OR = 1.27, 95% CI = 1.11–1.46, *p* = 0.017) and no significance of the reverse MR results (*p* > 0.05). Meta‐analysis of the MR results confirmed this finding and was significant in all population subgroups (OR = 1.29, 95% CI = 1.18–1.40). The findings were maintained after controlling confounders using MVMR (OR = 6.75, 95% CI = 1.50–30.44, *p* = 0.013). These analyses were confirmed to be reliable and stable by sensitivity testing.

**Conclusions:**

Our study found a positive causal effect of OA and AD, which was confirmed by the highest levels of evidence‐based medicine. It may provide meaningful evidence for the current controversy.

## Graphical abstract

Description of the design used in this Mendelian randomization (MR) study.

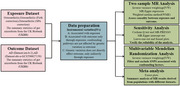

## 1 Introduction

Alzheimer's disease (AD) is the leading cause of chronic, progressive dementia, which is highly age‐related and one of the most common cognitive disorders in the elderly (Lauterborn et al. 2021; Plowey et al. 2022). Deposition of beta‐amyloid (Aβ) and phosphorylation of tau protein are the main pathological changes in its pathogenesis, but the pathogenesis is not clear (Hong and Lee 2017). AD is fast becoming one of the most costly, deadly, and burdensome diseases of this century (Scheltens et al. 2021). Approximately $226 billion was estimated to be spent on healthcare for Americans aged 65 and older with dementia in a 2015 survey. There are also a number of potential disease burdens that exist. More than 15.7 million family members and other unpaid caregivers provide approximately 17.9 billion hours of care to people with AD and other dementias, contributing $217.7 billion in value (Kirson et al. 2016). Therefore, searching for and identifying new causes of AD pathogenesis is a potential direction to reduce the burden of disease in AD patients.

Osteoarthritis (OA) causes damage to tissues such as articular cartilage, subchondral bone, and synovium, resulting in marginal osteophytes, and is one of the most common disabling diseases in the elderly (Yu et al. 2018). A study has predicted that OA will affect 595 million people globally in 2020, equivalent to 7.6% of the world's population, and represents a 132.2 percent increase in the total number of cases compared to 1990 (Minnig et al. 2024). A new study found a potential link between OA and AD. However, there is a lot of controversy surrounding this relationship. Heneka et al. found that chronic inflammation exists in AD patients and that overproduction of reactive oxygen species during the inflammatory process increases the level of reactive oxygen species in the body and puts the risk of OA at an increased level (Heneka et al. 2024; Zhao et al. 2020). However, Kyrkanides et al. (2011) found in a mouse model of OA that OA aggravates the development of neuroinflammation, which promotes the development of AD. In addition, current research is conflicting on the order of association between OA and AD. Understanding this relationship is beneficial for the prevention and treatment of OA and AD.

MR analyses can clarify the causal effect between exposure and outcome. It uses genetic variation as an instrumental variable (IV) for research and is unaffected by factors such as interference bias, confounders, and so forth. In this study, we examined the causal relationship between OA and AD and clarified the sequential aspects of this relationship. Furthermore, we hope to provide a high‐level evidence base in this area.

## 2 Materials and Methods

### 2.1 Data Source

GWAS data included in this study were obtained from a meta‐analysis published by the UK Biobank (UKBB) in 2021. All participants were European. A recent GWAS predicting OA‐associated single nucleotide polymorphisms (SNPs) was considered as exposure data and included a total of 484,598 OA patients (*N*_case_ = 39,515, *N*_control_ = 445,083) (Dönertaş et al. 2021). In addition, we included 407,746 patients from the Firth‐corrected OA population and 407,746 patients from the SPA‐corrected OA dataset population from a genome‐wide regression study in 2021 (Mbatchou et al. 2021). In summary, two GWAS datasets related to AD were considered outcomes. Specifically, AD (dataset: ebi‐a‐GCST90027158) included 487,511 cases of European origin (*N*_case_ = 39,106, *N*_control_ = 46,828), and AD (dataset: ieu‐b‐2) included 63,926 cases of males and females of European origin (*N*_case_ = 21,982. *N*_control_ = 41,944) (Bellenguez et al. 2022). We confirmed these data from the publicly available GWAS directory website (https://www.ebi.ac.uk/GWAS/downloads/summary‐statistics, Accessed January 10, 2024), and the data for this study were depersonalized so that patient consent was not required.

### 2.2 Selection of IVs

To ensure the accuracy and robustness of the causal relationship between OA and AD, we utilized the following steps to select IVs. In principle, the IVs used in MR analysis must satisfy the following three assumptions: (1) The IV must be related to exposure; (2) the IV must be associated with outcome only through exposure, with confounding pathways not affected by outcome genetic variation; (3) genetic variation does not directly affect results, but only indirectly through exposure (Xiao et al. 2022). First, SNPs with an association threshold of *p* < 5 × 10^−8^ were extracted for MR analysis to elucidate more considerable variations when few SNPs were available for exposure. Next, the independent variants were identified using the aggregation program implemented in R software (version 4.3.2), where the linkage‐imbalance threshold for *r* ^2^ < 0.001 within the 10,000 kb window of the European 1000 Genome Project Phase 3 reference panel. To emphasize the possibility of statistical bias in the raw GWAS, the effective allele frequency (EAF) threshold for IV should be > 0.01. Additionally, SNPs for moderate palindromic sequences that could not be adjusted were removed to ensure that the effects of the SNPs at the time of exposure were the same as the alleles corresponding to our results (Xu et al. 2022). And finally, we further quantified the strength of IV with the *F*‐statistic to facilitate the removal of SNPs that may lead to unwanted bias, where the *F*‐statistic > 10 as a valid tool provided reasonable evidence that IV was a powerful tool (Yang et al. 2023).

### 2.3 MR Analysis

We used a two‐sample MR analysis with genetic variation as an IV to initially assess the causal relationship between exposure (OA) and outcome (AD). The inverse variance weighting (IVW, random effect) method was used as the primary analysis method, and the weighted median method and MR‐Egger regression method were used as supplementary analysis methods. IVW is the most important method for assessing causality between exposure and outcome, based on the fundamental premise that all genetic variants are valid IVs and have a solid ability to detect causality (Xiao et al. 2022). An estimate of the IVW can be obtained by calculating the slope of the weighted linear regression. When there is no directed pleiotropy in SNPs, the IVW method is considered to be the most reliable (Ding et al. 2023). In addition, when up to 50% of the SNPs are derived from genetic variation in the null IV, the weighted median provides consistent estimates of causal effects (Xu et al. 2022). MR‐Egger regression can also be utilized to assess potential associations if the genetic variance is not valid. Ultimately, reverse MR analysis was performed using AD as the exposure and OA as the outcome, with the use of the same methods and settings described above (Wang et al. 2023).

### 2.4 Sensitivity Analysis

Since there was no pleiotropy hypothesis in MR, a sensitivity analysis was performed to verify the reliability of the analysis. First, Cochran's *Q* statistics were used to evaluate the heterogeneity of IVs, and the heterogeneity of the effect of SNPs related to three OA indicators on AD outcomes was detected (Yang et al. 2023). Initially, MR‐Egger was used to detect and correct for horizontal pleiotropy, which provides estimates consistent with the null instrument to determine whether the selected IVs have a multidirectional effect. MR‐Egger can detect violations of IV assumptions and provide impact estimates that are not affected by these violations. Furthermore, the intercept value in MR‐Egger is used to evaluate pleiotropy, and if the intercept is very close to 0, the MR‐regression model is very close to IVW (Xu et al. 2022). Afterward, horizontal multiplicity was detected and corrected with the Mendelian randomization pleiotropy residual sum and outlier (MR‐PRESSO) method. Finally, we used leave‐one‐SNP‐out analysis to identify potential influential SNPs and assess the reliability of the results. Funnel plots were used to evaluate the heterogeneity among SNPs (Xin et al. 2022).

### 2.5 Multivariable Mendelian Randomization Analysis

To filter and exclude SNPs associated with confounders and OA endings, we used the manual PhenoScanner database (http://www.phenoscanner.medschl.cam.ac.uk/ Accessed on January 10, 2024) to exclude exposure‐related instrumental SNPs from the resultant dataset. Phenotypic analysis of OA‐related SNPs identified confounders. We performed analyses by MVMR with OA as the primary variable and the remaining confounders as secondary variables, adjusting for confounders to determine whether confounders affect the causal relationship between OA and AD. Exposure‐related IVs were screened under the same two‐sample MR conditions, and confounders associated with significant two‐sample MR results (*p* < 0.05) were ultimately included in the MVMR analysis (Xin et al. 2022). Notably, SNPs highly associated with each exposure factor needed to be included in the joint set and matched to the results. Finally, when calculating the results, the predictions for each exposure were included in the multiple regression and evaluated using the IVW method (Kjaergaard et al. 2022).

### 2.6 Meta‐Analysis

In order to integrate all results derived from two‐sample MR, provide the highest level of evidence‐based medical evidence, and validate genetic findings, we employed meta‐analysis of the results of IVW for OA and AD. In addition, we performed subgroup analysis for OA and AD separately for populations originating from different databases. This procedure was carried out using STATA 12.0.

### 2.7 Statistical Analysis

All data analysis was performed using the “TwoSampleMR” and “MRPRESSO” packages of the R software (version 4.3.2). In two‐sample MR and MVMR, *p* < 0.05 indicated that the difference between the statistical tests was statistically significant. In contrast, in the test for heterogeneity, *p* > 0.1 indicated that there was no heterogeneity between groups (Lin et al. 2022). When the MR‐Egger regression intercept is not 0 and statistically significant (*p* < 0.05), horizontal pleiotropy of the IV is considered to exist (Xin et al. 2022). Reverse MR analysis (*p* > 0.05) showed no reverse causality between exposure factors and outcome variables.

## 3 Results

### 3.1 SNP Selection

**FIGURE 1:**
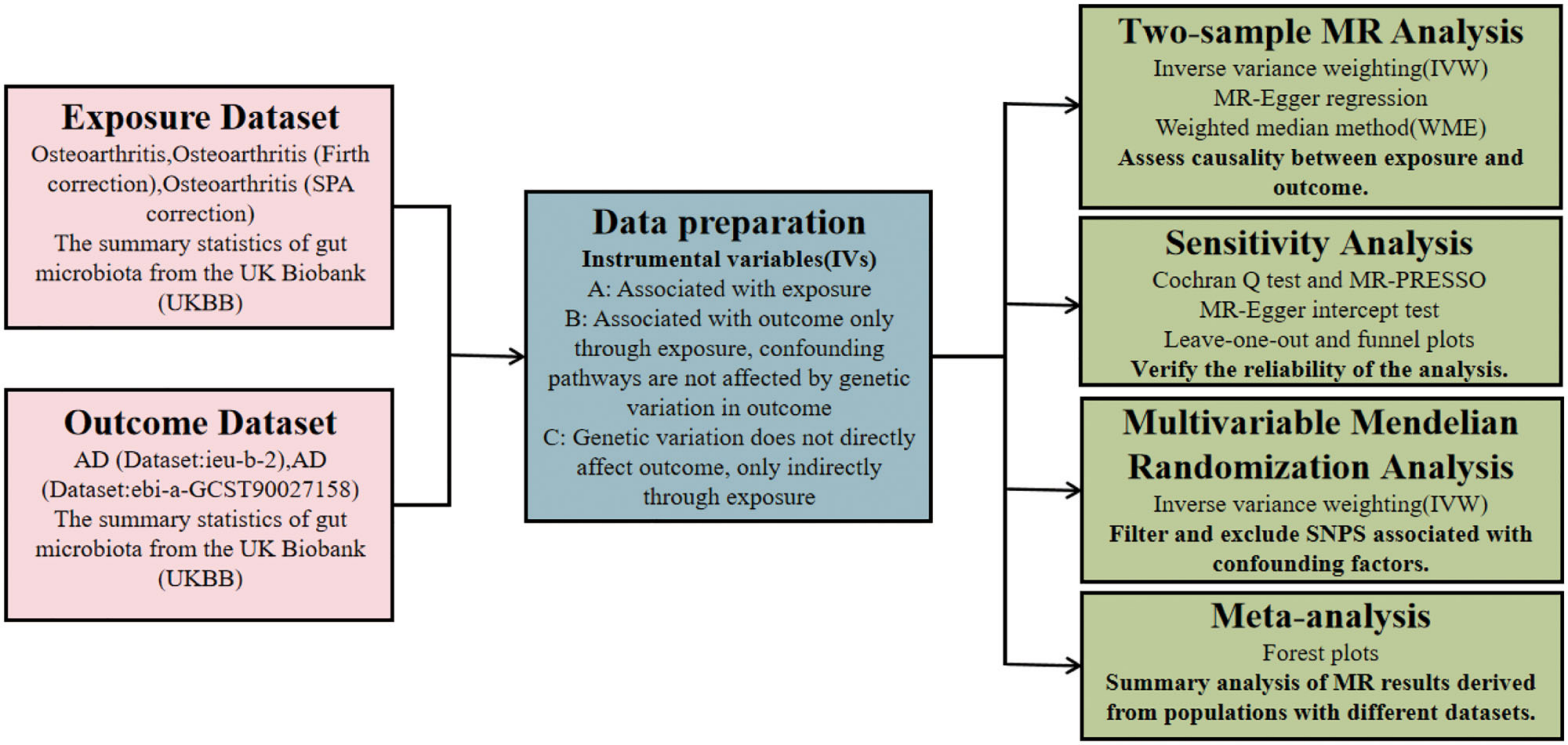
The flowchart of Mendelian randomization (MR).

Based on the description above, we selected independent SNPs associated with exposure. SNPs highly associated with AD were considered IV, and the *F*‐statistic of each SNP was greater than 10. After coordinating and excluding SNPs with moderate allele frequencies and palindromes, we finally extracted SNPs for OA, OA (Firth correction), and OA (SPA correction) with 6, 5, and 5 SNPs, respectively. Therefore, these significant SNPs were ultimately used as IVs for the two‐sample MR analyses of OA and AD (Figure 1).

### 3.2 Causality Between OA and AD

**FIGURE 2:**
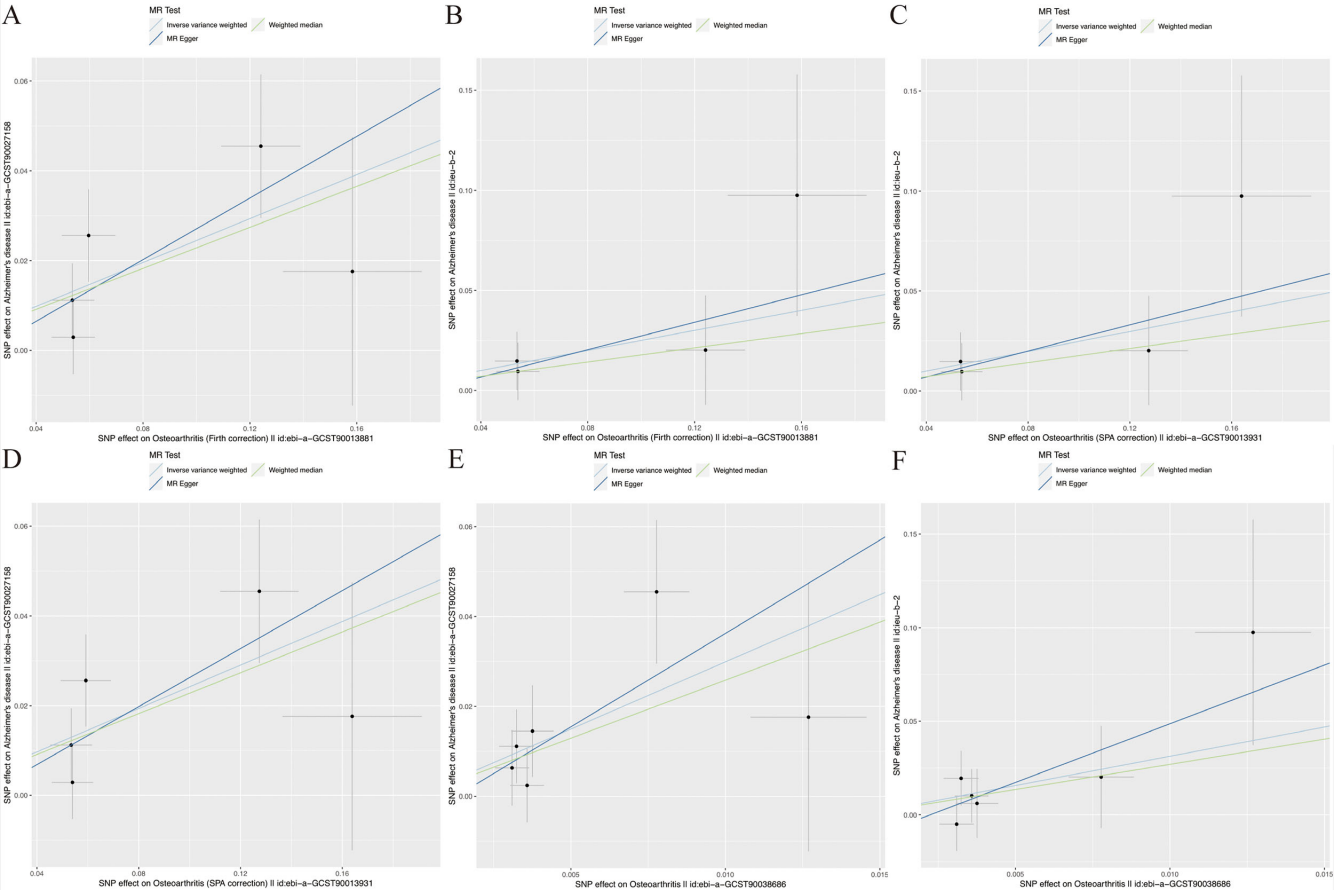
Scatter plots of two‐sample MR analysis of OA and AD. The horizontal axis represents the effect value of the exposure‐related SNP, and the vertical axis represents the effect value of the outcome. The colors and slopes of the straight lines represent the results from the three methods of IVW, MR‐Egger, and the weighted median method.

IVW analyses showed that OA was positively correlated with AD (dataset: ebi‐a‐GCST90027158). Specifically, when OA was utilized as the exposure and AD (dataset: ebi‐a‐GCST90027158) as the outcome, IVW results revealed positive correlations for OA, OA (Firth correction), OA (SPA correction), and AD (dataset: ebi‐a‐GCST90027158) (IVW OR = 19.89, 95% CI = 2.90–136.57, *p* = 0.002; OR = 1.28, 95% CI = 1.11–1.47, *p* = 0.017; OR = 1.27, 95% CI = 1.11–1.46, *p* = 0.017). The MR‐Egger results revealed OR > 1, consistent with the IVW results, indicating a positive correlation between exposure and outcome. After weighted median analysis, the results further confirmed a positive causal relationship between OA, OA (Firth correction), OA (SPA correction), and AD (dataset: ebi‐a‐GCST90027158) (*p* < 0.05, OR > 1). The results complemented the previous IVW results. However, when using AD (dataset: ieu‐b‐2) as the outcome and OA as the exposure, there was no significant difference between OA and AD (*p* > 0.05). Of note, our eventual reverse MR analysis did not reveal a statistically significant correlation (*p* > 0.05), and the conclusions were not meaningful (Table 1 and Figure 2).

**TABLE 1:** Results of the Causal Associations between OA and AD.

			IVW		Weighted median		MR‐Egger	
Exposure	Outcome	SNP (*n*)	*p*	OR (95%Cl)	*p*	OR (95% Cl)	*p*	OR (95% Cl)
OA (Firth correction) || ID: ebi‐a‐GCST90013881	AD || ID: ebi‐a‐GCST90027158	5	0.001	1.28 (1.11–1.47)	0.017	1.26 (1.04–1.51)	0.211	1.41 (0.92–2.16)
OA (SPA correction) || ID: ebi‐a‐GCST90013931	AD || ID: ebi‐a‐GCST90027158	5	0.001	1.27 (1.11–1.46)	0.017	1.26 (1.04–1.51)	0.218	1.38 (0.92–2.08)
OA || ID: ebi‐a‐GCST90038686	AD || ID: ebi‐a‐GCST90027158	6	0.002	19.89 (2.90–136.57)	0.043	13.23 (1.08–161.28)	0.169	63.83 (0.49–8283.48)
OA (Firth correction) || ID: ebi‐a‐GCST90013881	AD || ID: ieu‐b‐2	4	0.063	1.28 (0.99–1.67)	0.245	1.19 (0.89–1.61)	0.428	1.41 (0.71–2.78)
OA (SPA correction) || ID: ebi‐a‐GCST90013931	AD || ID: ieu‐b‐2	4	0.062	1.28 (0.99–1.66)	0.258	1.19 (0.88–1.62)	0.427	1.39 (0.73–2.65)
OA || ID: ebi‐a‐GCST90038686	AD || ID: ieu‐b‐2	6	0.074	22.82 (0.73–708.90)	0.223	14.82 (0.19–1129.32)	0.246	529.75 (0.06–4473945.81)

### 3.3 Sensitivity Analysis

**FIGURE 3:**
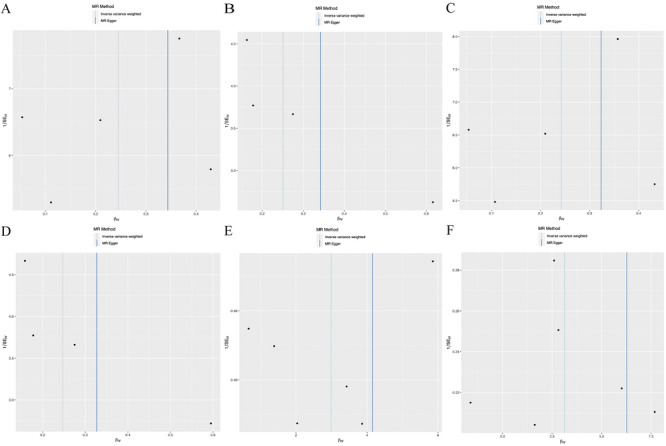
Funnel plots were used to visualize IVs’ sensitivity. A black dot represents an SNP, and a light‐colored vertical line represents the IVW method.

**FIGURE 4:**
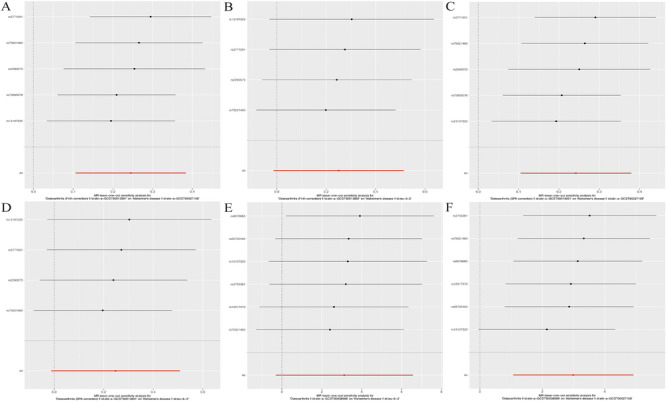
Results of the leave‐one‐out in MR analysis were used to determine the stability of the results. Each line above represents the results of the MR analysis after the deletion of SNPs, and the line below represents the results of the entire MR analysis.

A sensitivity analysis was performed to verify the reliability of the test results further. The IVW test for heterogeneity showed that there was no difference in the heterogeneity results compared with those of the MR analyses between OA and AD (*p* > 0.05). Horizontal pleiotropy between IV and outcomes was assessed using the intercept test of MR‐Egger regression analysis, which showed weak evidence of horizontal pleiotropy. Similarly, the MR‐PRESSO assay revealed no horizontal pleiotropic effect of abnormal SNPs on the risk of OA (*p* = 0.596), OA (Firth correction) (*p* = 0.442), and OA (SPA correction) (*p* = 0.451) (Table 2). Cochran's *Q* statistic showed that there was no heterogeneity in IV. In addition, based on the symmetry of the funnel plot, which showed no potential heterogeneity among genetic variants, horizontal pleiotropy was not significant (Figure 3). The leave‐one‐out algorithm indicated that no single SNP strongly influenced the potential causal relationship between OA and AD in the exclusion of single SNP analyses, suggesting that the results of the current MR analyses are robust (Figure 4).

**TABLE 2:** MR Estimates for the Association Between OA and AD.

		Heterogeneity		MR‐Egger regression			MR‐PRESSO
Exposure	Outcome	MR‐Egger	IVW	Intercept	SE	*p*	*p*
OA (Firth correction) || ID: ebi‐a‐GCST90013881	AD || id:ebi‐a‐GCST90027158	0.277	0.384	−0.007	0.015	0.659	0.442
OA (SPA correction) || ID: ebi‐a‐GCST90013931		0.269	0.385	−0.006	0.014	0.702	0.451
OA || ID: ebi‐a‐GCST90038686		0.490	0.596	−0.005	0.010	0.636	0.596
OA (Firth correction) || ID: ebi‐a‐GCST90013881	AD || id:ieu‐b‐2	0.583	0.762	−0.007	0.024	0.801	0.808
OA (SPA correction) || ID: ebi‐a‐GCST90013931		0.584	0.767	−0.006	0.024	0.818	0.814
OA || ID: ebi‐a‐GCST90038686		0.745	0.778	−0.014	0.019	0.502	0.815

### 3.4 Multivariable Mendelian Randomization Analysis

In the PhenoScanner database, we identified confounders of OA, including stroke, body mass index (UKB data field 21001), hypertension, and diabetes diagnosed by doctors. Next, we separately calculated, using two‐sample MR, the confounding factors between the causal relationships. The results after removing the covariate exposure revealed that there was a significant causal relationship between body mass index (UKB data field 21001), hypertension (OR = 0.87, 95% CI = 0.80–0.94; OR = 0.59, 95% CI = 0.47–0.74), and AD. We then included the above positive results in the subsequent analysis of the MVMR method to adjust for the effect of confounder inclusion on the results. After adjusting for confounders using IVW, OA remained positively correlated with AD (OR = 6.75, 95% CI = 1.50–30.44, *p* = 0.013) (Table 3).

**TABLE 3:** Results of MVMR Analysis.

Exposure	*p*	OR (95% Cl)
Body mass index (UKB data field 21001)	0.0004	0.87(0.80–0.94)
Hypertension	< 0.0001	0.59(0.47–0.74)
Osteoarthritis	0.0130	6.75(1.50–30.44)

### 3.5 Meta‐Analysis

**FIGURE 5:**
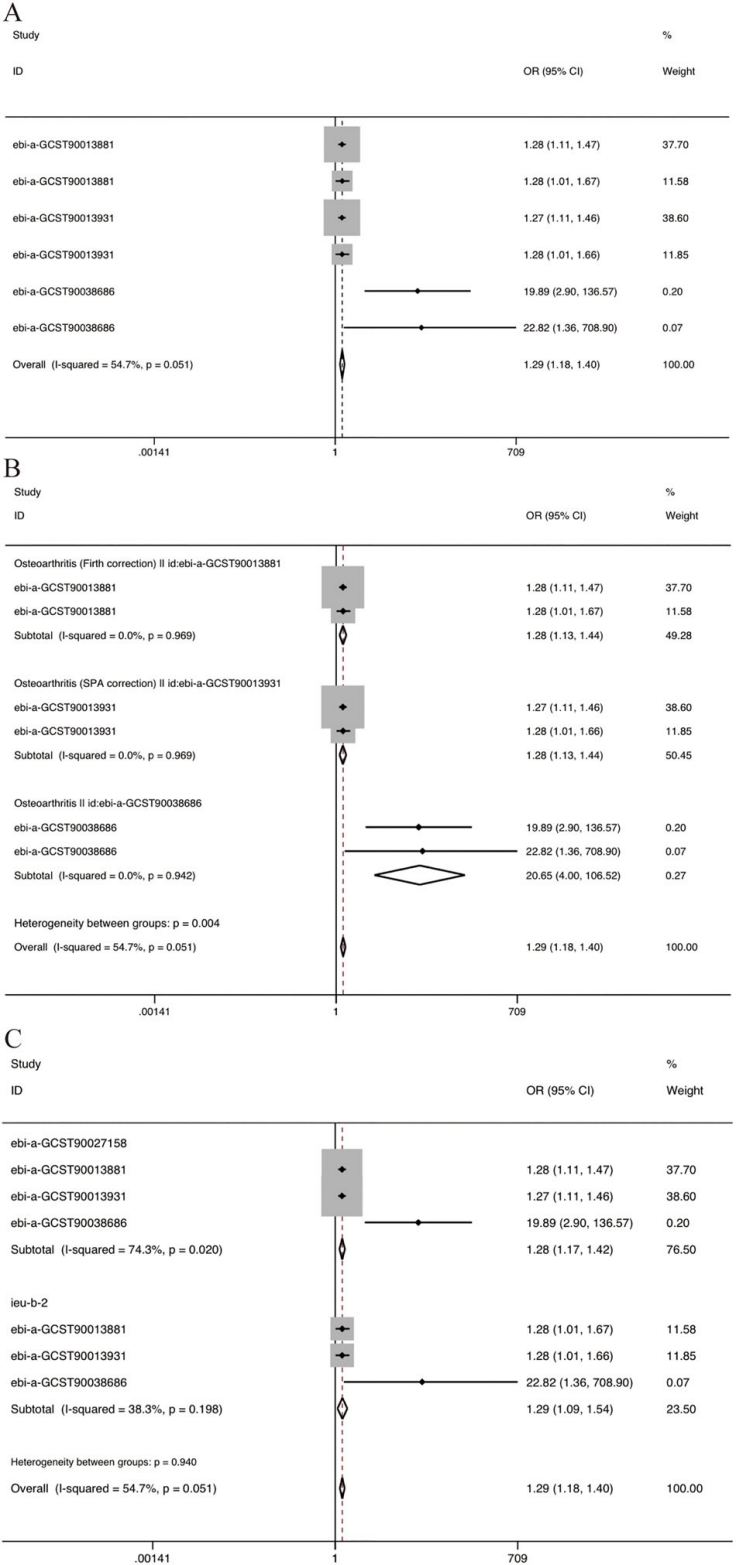
Forest plots for meta‐analysis. (A) Meta‐analysis based on MR results. (B) Subgroup analysis based on the OA population from different databases. (C) Subgroup analysis based on AD populations from different databases.

We conducted a meta‐analysis of the results of the two‐sample MR and indicated that the overall effect of OA on AD was contributory (OR = 1.29, 95% CI = 1.18–1.40). When results from AD populations originating from different datasets were subgroup analyzed, we found that the final results were all positively correlated. Interestingly, although the AD (dataset: ieu‐b‐2) population showed negative MR results in the two‐sample MR, a subgroup analysis of it revealed a positive correlation with OA (OR = 1.29, 95% CI = 1.09–1.54). In addition, the results of the OA population originating from different datasets were analyzed by subgroup and also showed a positive correlation (Figure 5).

## 4 Discussion

In this study, we performed an unbiased assessment of the causal relationship between OA and AD using different GWAS datasets. We utilized two‐sample MR to analyze the causal relationship between OA and AD at the gene level, effectively avoiding the influence of confounders in traditional observational studies (Ding et al. 2023). The MR results showed that OA (dataset: ebi‐a‐GCST90027158) was significantly and positively associated with AD at the gene level. Noticeably, although there was no relationship between OA (dataset: ieu‐b‐2) and AD, we performed a meta‐analysis to merge the results derived from different sources of population data and finally concluded that the total effect of OA on AD was still positively correlated, which provided us with the most substantial evidence‐based medical evidence. In further studies, sensitivity analyses were performed to ensure the accuracy of the results. In addition, confounding factors could be excluded using MVMR, and the causal effect of AD on OA could be excluded by reverse MR. In conclusion, our study found that OA had a contributory effect on AD, which provided practical insights for studying the pathogenesis and prevention of AD.

Currently, more and more studies are trying to verify the relationship between OA and AD, but the relationship between the two is highly controversial. Although some studies have shown that OA may accelerate the onset of AD (Du et al. 2023; Kalvaityte et al. 2022; Lluch et al. 2014), another study of the influences exerted by uric acid in the development of AD progression revealed that people who had a previous history of OA had a 24% lower risk of developing AD, which may be attributed to the potentially neuroprotective effects of uric acid, and these effects lead to the suppression of AD onset by OA (Lu et al. 2016). In addition, the upstream and downstream relationships of OA and AD are not clear. Several studies have shown that OA accelerates the onset and progression of AD (Gupta et al. 2023; Huang et al. 2015; Kyrkanides et al. 2011). However, some studies have opposing views. For example, AD patients were found to have upregulated miRNA‐29b in their bodies, which may further promote chondrocyte apoptosis and induce OA (Gentile et al. 2022; Iulian Stanciugelu et al. 2022). Therefore, we thought it was necessary to examine the controversial issues between OA and AD further in order to clarify the link between them. Interestingly, Cai et al. (2021) explored the causal relationship between OA and AD using MR methods in 2013 but failed to find a meaningful causal relationship between OA and AD. We included in our study newer data that included a larger and broader population for a two‐sample MR analysis, which showed a positive causal relationship between OA and AD. In addition, we utilized MVMR, reverse MR, sensitivity analysis, and meta‐analysis to provide stronger evidence for the conclusions of two‐sample MR. Therefore, we consider their previous conclusions unreliable.

A number of novel therapeutic strategies have emerged for OA and AD, significantly enhancing disease outcomes (Table 4). For instance, a disease‐modifying osteoarthropathy drug (DMOAD) under development has proven effective in decelerating the degeneration of articular cartilage (Cho et al. 2021). Additionally, stem cell therapy has demonstrated potential in the OA field, as the exosomes it generates can promote cartilage and bone regeneration, playing a crucial therapeutic role (Song et al. 2021). In the realm of AD, nanoparticle‐based therapies are emerging as cutting‐edge treatment options. These therapies can cross the blood‐brain barrier (BBB) and enhance drug bioavailability (Agraharam et al. 2022; Harini et al. 2022). Some animal‐derived biomolecules, like lumbrokinase (LK), have also been shown to have the potential to degrade Aβ 1–42 amyloid and could be suitable candidates for AD treatment (Metkar et al. 2024). Moreover, immunotherapy has shown promising applications in AD treatment by modulating the host immune system, which underlines the important role of immunomodulation in neurodegenerative disease (Balkhi et al. 2025). Our study systematically validates the association between OA and AD. From a comorbidity perspective, it thereby reveals the upstream–downstream relationship between the two diseases in terms of pathological mechanisms. This finding offers a theoretical basis for the development of more effective therapeutic strategies from a multi‐disease interaction perspective and presents new treatment ideas for patients with comorbid OA and AD.

**TABLE 4:** Recent emerging therapeutic strategies for OA and AD.

Disease	Author	Therapeutic strategy	Main Outcomes
OA	Kim et al.	DMOAD	DMOAD can mitigate the degeneration of articular cartilage in osteoarthritis by specifically targeting inflammatory cytokines, matrix‐degrading enzymes, and the Wnt pathway (Cho et al. 2021)
	Liang et al.	ADMSC‐derived exosomes	Exosomes from MSCs facilitate progenitor and stem cell differentiation into mature chondrocytes, enhancing cartilage and bone regeneration, and yielding therapeutic benefits in OA treatment (Song et al. 2021)
	Klein et al.	Hydration lubrication	Intra‐articular injection of liposomes can reduce friction on the articular cartilage surface. Additionally, they can be utilized as controlled‐release anti‐inflammatory or analgesic agents, thereby effectively treating OA (Lin and Klein 2022).
	Wu et al.	Gene therapy	The gene therapy vector, Adeno‐associated virus (AAV), can co‐deliver IL‐1Ra and SOX9 to inhibit IL‐1‐mediated inflammatory signaling, maintain cartilage homeostasis, and delay the progression of OA (Zhou et al. 2025).
	Xu et al.	miRNA	miR‐199a‐3p can mediate the mTOR autophagy pathway to enhance anabolic processes and inhibit catabolism in OA cartilage, thereby promoting cartilage repair (Zhao et al. 2023)
AD	Khan et al.	Nanocomposites	Nanocomposites loaded with Au nanoparticles exhibit therapeutic potential for AD by interfering with amyloid‐forming enzymes to reduce amyloidogenesis (Rajkumar et al. 2025)
	Colonna et al.	Immunotherapy	mAb‐mediated hTREM2 can inhibit neurotoxic Aβ plaques and neuronal damage, thereby achieving therapeutic benefits in AD (Wang et al. 2020).
	Kou et al.	Lifestyle intervention	Diverse exercise modalities can mitigate brain aging and decrease the accumulation of AD‐associated pathological proteins through the upregulation of Notch signaling and autophagy pathways, thereby exerting therapeutic efficacy in AD (Chen et al. 2025)
	Griffioen et al.	ReS19‐T	ReS19‐T binds to septins to inhibit pathological SOCC activation, restoring calcium homeostasis and mitigating β‐amyloid plaque and hyperphosphorylated tau aggregate formation, thereby protecting against AD (Princen et al. 2024).
	Arnold et al.	Insulin‐sensitizing medicine	Metformin exerts beneficial impacts on AD by augmenting orbitofrontal cortex metabolism, thereby enhancing executive and cognitive functions in AD patients (Koenig et al. 2017).

Several studies of potential biological mechanisms have also found that OA can contribute to the progression of AD. For one, β‐amyloid deposition, neurodegeneration, and neuroinflammation might be essential factors in accelerating and exacerbating the pathologic changes of AD in OA (Kyrkanides et al. 2011). Gupta et al. (2023) found in their study on AD–OA mice that OA significantly increased β‐amyloid deposition and neuronal loss in AD mice, which accelerated the deposition of amyloid plaques in AD–OA mice and neurodegeneration, suggesting that OA is a risk factor for AD. It has also been found that the specific mechanisms by which peripherally induced neuroinflammation is associated with AD pathology may include increased Aβ production, decreased Aβ catabolism, or altered Aβ transport and that neuroinflammatory signals may limit the ability of microglia and other cells to clear Aβ plaques (Hickman et al. 2008; Jaeger et al. 2009; Sheng et al. 2003). At the same time, since OA is associated with significantly elevated levels of the pro‐inflammatory cytokines in the blood, the progression of AD may thereby be exacerbated. Mechanisms have been identified that blood levels of CRP reflect localized joint inflammation in patients with advanced OA and that AβIL‐1 and CRP are important risk factors for the development of AD. AβIL‐1 is also thought to drive the production of β‐amyloid precursor protein, which regulates the deposition of amyloid plaques in the brain of AD patients (Al‐Khazraji et al. 2018; Innes and Sambamoorthi 2020; Lluch et al. 2014). Supplementarily, the gene for cluster protein, a biomarker of early OA, has been identified as an influential risk locus for AD (Desikan et al. 2014; Kalvaityte et al. 2022). Several studies have reported that CLU interacts with and effectively inhibits the aggregation of TDP‐43 and that CLU also reduces the number of cytoplasmic TDP‐43 inclusion bodies in a model of neuronal cells under ER stress. Thus, the reduced secretion of clusterin in OA patients is a mechanism for the associated CLU mutations in AD patients (Bettens et al. 2015). All these studies mentioned above provide a theoretical basis for our conclusions and provide evidence for the biological mechanisms by which OA leads to the progression of AD.

Our study has various strengths. Firstly, we used a two‐sample MR study to identify potential causal associations between OA and AD. To improve the study's accuracy, we excluded the influence of external confounders and used reverse validation to ensure the unidirectionality of the causal association. Secondly, we utilized the large‐scale UKBB database, which offered a substantial sample for the GWAS analysis (Xin et al. 2022). We excluded nonoverlapping and independent exposure and outcome data to ensure the robustness and scope of the assessment (Wang et al. 2023). Furthermore, we performed sensitivity analyses to test the reliability of our findings (Lin et al. 2022). Finally, we conducted a cumulative meta‐analysis of studies from different databases to test the stability and adequacy of evidence accumulation.

Of course, our study has some limitations. Firstly, our study only analyzed populations from Europe, and the generalizability of use when extending our findings to other populations needs to be clarified (Xu et al. 2022). Second, the two‐sample MR explored a linear relationship between exposure and outcome and did not allow for a nonlinear analysis of exposure and outcome. Third, our study was conducted only at the genetic level and did not examine whether specific targets and metabolites in patients with OA have a dramatic effect on the development and progression of AD (Yang et al. 2023). Fourth, the data from our study lacked individualization and could not be stratified by categories such as gender (Ding et al. 2023). Therefore, the two‐sample MR analysis allowed only a preliminary judgment of causality, and the specific biological mechanisms deserve further investigation.

## 5 Conclusion

Based on the publicly available GWAS database, our study demonstrated a positive causal relationship between OA and AD from the perspective of genetic variation by two‐sample MR, MVMR, reverse MR, and meta‐analysis. Our study provided the highest evidence‐based medical evidence of causality for the relationship between OA and AD, presenting clear proof of the controversy that exists between OA and AD. Identification of potential risk factors for OA and treatment in a timely manner is constructive in reducing the incidence of AD.

## Author Contributions

**Jingkai Di**: writing – original draft, methodology, conceptualization. **Yujia Xi**: writing – review & editing, validation. **Yaru Liu**: writing – review and editing. **Likun Qi**: writing – review and editing. **Tingting Chen**: writing – review and editing. **Shuai Chen**: writing – review and editing. **Chuan Xiang**: writing – review and editing, methodology, funding acquisition, formal analysis, data curation, conceptualization.

## Conflicts of Interest

The authors declare no conflicts of interest.

### Peer Review

The peer review history for this article is available at https://publons.com/publon/10.1002/brb3.70455

## Data Availability

The data that support the findings of this study are openly available in GWAS at https://www.ebi.ac.uk/gwas/
